# Nursing students’ perspectives on implicit rationing of nursing care: Cross-sectional and cross-cultural analysis

**DOI:** 10.1016/j.ijnsa.2026.100544

**Published:** 2026-04-29

**Authors:** Havva Arslan Yürümezoğlu, Milena Marta Bruschini, Maria Schubert

**Affiliations:** a Dokuz Eylul University, Faculty of Nursing, Department of Nursing Management, İzmir, Türkiye; b ZHAW Zurich University of Applied Sciences, School of Health Sciences, Institute of Nursing, Winterthur, Switzerland

**Keywords:** Health care rationing, Nursing students, Nursing care, Cross-sectional studies, Cross-cultural comparison

## Abstract

**Background:**

Scarce or insufficient nursing resources are an important reason that nurses have to ration necessary nursing tasks implicitly. Implicit rationing of nursing care is associated with negative patient and nurse outcomes, such as poor patient safety and low job satisfaction. The frequency at which nursing students implicitly ration nursing tasks during their clinical placements and the prioritisation strategies they use remain unclear.

**Aim:**

To describe the levels of implicit rationing of nursing care, prioritisation strategies used by Swiss and Turkish nursing students during their clinical placements, and characteristics of their nursing work environment.

**Methods:**

This descriptive, cross-sectional observational study included 506 Swiss and 750 Turkish eligible students from four universities, with a final sample size of 81 and 205 students, respectively. Implicit rationing of nursing care was measured using the Basel Extent of Rationing of Nursing Care-Revised (BERNCA-R) version on a scale of 0 (‘never’) to 3 (‘often’). The prioritisation strategies were assessed with a single item. In addition, data on workplace characteristics and demographics were collected. Data were analysed using descriptive statistical methods.

**Results:**

Among the 23 BERNCA-R items compared in this study, the reported rationing levels in the Swiss sample ranged from 0.52 (‘change of wound dressings’) to 1.83 (‘emotional and psychological support’), and in the Turkish sample, the levels ranged from 0.74 (‘necessary disinfection measures’) to 1.63 (‘sponge bath’). The Swiss students prioritised nursing tasks primarily based on ‘their necessity’ and/or ‘associated consequences for patient safety’, whereas the Turkish students prioritised them mainly based on ‘their necessity’. The Swiss students rated the staffing and resource adequacy, overall quality of the work environment, quality of care, and patient safety at their most recent clinical placement workplace slightly higher than that rated by the Turkish students.

**Conclusions:**

This study demonstrates the importance of teaching resource scarcity management and care prioritisation strategies to reduce students' implicit care rationing during clinical placements.

**Tweetable abstract:**

Implicit rationing and associated factors from the perspective of nursing students: a cross-sectional and cross-cultural comparative observational study, from Switzerland and Türkiye.

**What is already known**

• Rationing of care is a widespread issue in nursing practice and clinical nursing education.
• Students develop a significant part of their professional socialisation through interactions with colleagues during clinical practice.
• The nursing work environment that students encounter during clinical practice plays a crucial role in shaping their perspectives on the rationing of nursing care.

Alt-text: Unlabelled box dummy alt text

**What this paper adds**

• Swiss nursing students predominantly rationed nursing tasks related to psychosocial and rehabilitative care, whereas Turkish students were more likely to ration physical and basic daily care tasks.
• The majority of Swiss and Turkish nursing students prioritised nursing tasks based on necessity.
• Although Swiss students perceived the safety and quality of their practice work environment more positively than Turkish students, both groups rated it as moderate overall.

Alt-text: Unlabelled box dummy alt text

## 1 Introduction

The failure to provide necessary patient care owing to insufficient nurse staffing and limited resources presents a serious patient safety issue (Assaye et al., 2022; Hessels et al., 2019). Different overlapping terms have been used in the literature to describe this issue, such as 'unfinished care', 'care left undone', 'omitted care', 'missed care', 'rationing of care', and 'implicit rationing of nursing care' (Jones et al., 2015). Missed nursing care is an umbrella term encompassing any aspect of required care that is left incomplete owing to insufficient resources, patient-related barriers or delays, or nurse neglect or delay (Kalisch et al., 2009). Contributing factors to missed nursing care include nurse staffing levels, lack of appropriate skill mix and material resources, patient acuity, and deficiencies in teamwork or communication (Bagnasco et al., 2020; Chaboyer et al., 2021). Missed nursing care negatively impacts patient outcomes, including reduced quality of care, lower patient satisfaction, increased adverse events (e.g., falls, pressure ulcers), longer hospital stays, and higher readmission rates (Chaboyer et al., 2021; Recio‐Saucedo et al., 2018). Moreover, it has been associated with greater job dissatisfaction, burnout, and an increased intention to leave the profession (Stemmer et al., 2022).

This study focuses specifically on implicit rationing of nursing care, a subcategory of the broader concept of missed care. In this study, implicit rationing refers to the withholding of or failure to carry out the necessary measure for patients due to lack of nursing resources, such as staffing, skill mix, or time (Schubert et al., 2005). Within this definition, necessary nursing measures refers to a set of nursing or treatment tasks that are accepted as important for a patient to achieve the desired outcomes. This phenomenon may be influenced by professional standards, nurses’ educational levels, and the cultural context of the country (Schubert et al., 2007). A systematic review found that 75% or more of the nurses were unable to complete at least one necessary nursing task during their most recent shift owing to time constraints (Griffiths et al., 2018). The most commonly rationed aspects of nursing care include communication and information sharing, support for self-management and autonomy, patient education and care planning, discharge planning and decision-making, basic physical care, and emotional and psychological care (Bagnasco et al., 2020; Chaboyer et al., 2021).

Implicit rationing of nursing care is a significant issue that extends beyond the clinical setting into the realm of nursing education. Nursing students acquire much of their professional socialisation during clinical practice from the behaviours and norms of the healthcare environment. Therefore, the nursing care environment students that encounter influences their perspectives on implicit rationing of nursing care. Consequently, they may become socialized to an environment, in which implicit rationing of nursing care is perceived as a normative, remains underreported, and its implication for patient outcomes receive insufficient attention. When unmet care needs are routinely accepted, students tend to pragmatically normalise this phenomenon rather than critically questioning or addressing its underlying determinants, which are not necessarily attributable solely to care rationing (Gibbon and Crane, 2018). Reported rates of care rationing are higher in the early years of nursing education and tend to decline towards graduation (Palese et al., 2023). However, most nursing education programs continue to emphasise a biomedical model of care, which reinforces a service-oriented view of nursing centred on physician priorities (Kalánková et al., 2021). A qualitative study in Germany found that nursing students often felt unable to meet professional standards of care in hospitals, perceiving themselves as ‘assembly-line workers’ tasked with fulfilling only basic patient needs (Habermann et al., 2022).

Within the framework of the COST Action RANCARE CA15208 – Rationing – Missed Nursing **Care**: An **I**nternational and **M**ultidimensional **P**roblem (2016–2021) (Papastavrou and Lemonidou, 2021), several national and international research projects were conducted or initiated in subsequent years, such as the studies by Habermann et al., 2022; Kalánková et al., 2021; Palese et al., 2021. Building on this foundation, members of the COST Action from Switzerland, together with associated partners from Türkiye, decided to conduct a study focusing on nursing students.

The aim of the study was to describe the levels of implicit rationing of nursing care and the prioritisation strategies used, from the perspective of Swiss and Turkish nursing students during their clinical placements, as well as the characteristics of their nursing work environments.

## 2 Research questions

1. Are there differences in the levels of rationing reported by nursing students between the two countries (Switzerland, Türkiye) involved?
2. How do nursing students prioritise the necessary nursing tasks in situations when they are unable to carry out all the required tasks for all their patients?
3. How do nursing students in Switzerland and Türkiye perceive the adequacy of nurse staffing, resources and patient safety in the clinical practice environment?

## 3 Methods

### 3.1 Design

This international study was conducted using a cross-sectional observational design. Building on the previous study, which descriptively reported on the clinical learning environment and the frequency of implicitly rationed nursing care using data from Türkiye only (Arslan Yürümezoğlu, 2024), this article broadens the scope to include a second country, presenting comparative analyses across of both.

### 3.2 Participants and setting

The sample consisted of nursing students enrolled in the bachelor’s program at four universities located in Switzerland and Türkiye (three universities of applied sciences in Switzerland and one university in Türkiye). Bachelor students from the four participating universities were included in the study if they were in clinical placement at the time of the survey or had completed clinical placement no more than one month previously. The population consisted of 516 students in Switzerland and 750 students in Türkiye. In Switzerland, 101 students opened the information letter and consent forms; eight students decided not to participate, and 12 provided incomplete answers, leaving a total of 81 students (response rate 15.7%) who were included in the analysis. In Türkiye, 357 students opened the questionnaire; however, 152 provided incomplete answers, leaving a total of 205 students (response rate 27.3%) who were included in the analysis. The students in Switzerland and Türkiye do not work completely independently. Depending on the semester of education and educational setting, they are supported by instructors, preceptors, trainers or supervisors in both countries.

**Variables and Measurements**: Implicit rationing of nursing care was measured with the Basel Extent of Rationing of Nursing Care-Revised (BERNCA-R) (Schubert et al., 2013). The PES-Nursing Work Index Subscale “Staffing and Resources Adequacy” (Lake, 2002) was used to measure the four characteristics of the nursing work environment. In addition, questions about demographic characteristics such as age, sex, academic year, and satisfaction with the choice of profession were also included. All primary and secondary endpoints and their measurements are listed in Table 1.

**Table 1:** Description of the study variables, instruments, and data sources.

Variable	Definition	Instrument and sources	
Primary endpoints			
Implicit rationing of nursing care	The extent of implicit rationing of nursing care reported by nursing students, the frequency of necessary nursing tasks for patients that were withheld or not performed in the last seven working days, due to a lack of time, staffing levels and/or skill mix.	Rationing was measured using the BERNCA-R (Schubert et al., 2013), which is an adapted version of the BERNCA-R developed by (Schubert et al., 2007). In Switzerland the BERNCA-R with 32 items was used, which contains a 6-point Likert-type scale (0=never, 1=rarely, 2=sometimes, 3=often, 4=not required, 5=not part of my tasks (Schubert et al., 2013). In Türkiye, the culturally adapted BERNCA-R scale was used, which contains 27 items and a 4 point- Likert-type Scale (0=never, 1=rarely, 2=sometimes, 3=often, 4=not required, 5=family member did) (Arslan Yürümezoğlu et al., 2023). Students rated how often they had to ration the listed tasks in their last seven working days. The BERNCA-R has a good construct and criterion validity, and reliability. The Cronbach’s Alpha of the Swiss version is 0.94 and of the Turkish version 0.91 (Arslan Yürümezoğlu et al., 2023; Schubert et al., 2013).	
Secondary endpoints			
Comparability of the amount of rationed care	Comparability of the reported amount of rationed nursing tasks	Single item. Students indicate (1=comparable, 2=lower, 3=higher) whether the amount of implicit rationed nursing care in their most recent shift was comparable to that of other shifts.	
Prioritisation strategies	Nursing students self-reported strategies which they used to prioritise the necessary nursing tasks	Single item, developed for this study. Students were asked to indicate which of the five listed strategies (1= necessity, 2= consequences for patient safety, 3= rules/strategies learned, 4= rules current workplace, 5= other strategies) they used to prioritise the necessary nursing tasks in situations when they are unable to provide all necessary care to all patients. Students only had to answer this question if they had indicated in the BERNCA questionnaire that they were unable to perform all the necessary tasks for each of their patients.	
Characteristics of the most recent workplace			
Nurse-to-patient ratio	Number of patients the students were directly responsible for on their most recent shift	Single item, adapted from previous studies (Sermeus et al., 2011). Students indicated the number of patients they were responsible for on their most recent shift, ranging from 1 to 30.	
Staffing and resource adequacy	Adequacy of staffing and resources in terms of enough time, enough nurses (RN), enough staff to get the work done, and adequate support service	Staffing and resources adequacy subscale of the Practice Environment Scale of the Nursing Work Index, with four items (PES-NWI-R, Lake, 2002). Using a 4-point Likert-type scale (1= strongly disagree to 4= strongly agree), students indicated their agreement with each of the four items (enough time to discuss patient care problems, enough nurses to provide high-quality care, enough staff to get the work done, and adequate support service). The reliability, Cronbach’s Alpha, of this subscale varied from 0.71–84 (Lake, 2002).	
Overall quality of the nursing work environment	Nursing students’ perceptions of the quality of the nursing work environment	Single item, adapted from previous studies (Sermeus et al., 2011). Students rated on a Visual Analogue Scale (VAS) (0=poor to 10=very good) the overall quality of the nursing work environment in the ward in which they recently worked.	
Patient safety	Nursing students' perceptions of patient safety	Single item, adapted from previous studies (Sermeus et al., 2011). Students rated on a VAS scale (0= poor to 10= very good) the overall grade of patient safety, in the ward they recently worked	
Quality of care	Nursing students’ perceptions of the quality of care provided to patients	Single item, adapted from previous studies (Sermeus et al., 2011). Students rated on a VAS scale (0=poor to 10=very good) the overall quality of care in the unit in which they recently worked.	
Demographics			
Age	Age of the student in years	Single item. Students indicated their current age in years.	
Sex	Sex of the student	Single item. Students indicated their sex (female or male).	
Academic year	Academic year of the student	Single item. Students indicated their academic year (first, second, third, or fourth)	
Career satisfaction	Satisfaction with the choice of a nursing career	Single item, adapted from previous studies(Sermeus et al., 2011). Students indicated on a 4-point Likert-type scale (1 = very dissatisfied to 4 = very satisfied) their career satisfaction	

### 3.3 Data collection

The data were collected in both countries between April and December 2021 using an online questionnaire (Swiss and Turkish versions). In Türkiye, nursing students accessed the questionnaire via a link provided through the Sakai Learning Management System, whereas in Switzerland, it was made available via a link on the Moodle Learning Management System. Study data were recorded electronically using the secure software, Research Electronic Data Capture (REDCap) (Harris et al., 2019, 2009). The electronic Case Report Forms were generated with REDCap. Only members of the research team had access to the data in REDCap.

### 3.4 Data analysis

REDCap automatically generated a data frame. Data analysis was conducted using IBM SPSS Statistics for Windows (Version 20.0.1.0). Only participants who answered at least half of the BERNCA-R items (11/23) were included in the analysis. Missing data were not replaced.

Descriptive statistics, including frequencies, ranges, means and standard deviations, were used to describe the levels of implicit rationing of nursing care reported by nursing students, the strategies they use to prioritise the necessary nursing tasks, and the role of the work environment. In the analysis included were the 23 BERNCA-R items to which data were collected in both countries (Switzerland 23/32, Türkiye 23/27). For calculating the mean values of the BERNCA-R, responses on a scale of 0–3 (0 = never, 1 = rarely, 2 = sometimes, 3= often) were included. Reponses of 4 (not required) and 5 (not part of my tasks), were excluded from the mean value calculation because they did not indicate an evaluative judgment but rather indicated the absence of a care requirement or the respondents’ lack of authority/responsibility owing to differences in students’ clinical practice settings and objectives. Higher BERNCA-R mean values indicated that the tasks were more frequently rationed.

### 3.5 Ethical considerations

Permission to conduct the study was requested from the responsible ethics committees. In Switzerland, a declaration of non-responsibility was provided by the cantonal ethics committee (Req-2021–00,624), as the study does not fall within the scope of the Human Research Act. In Türkiye, approval was obtained from the Nonclinical Ethics Committee (Req-2021/19–55). There was no risk to the participants involved in this study. Participants were informed that the information they provided would remain confidential, no personal identifiers would be collected, and all data would be recorded anonymously. Students were informed at the beginning of the questionnaire that by completing the survey, they were giving their consent for the data to be analysed as part of the study.

## 4 Results

### 4.1 Sample characteristics

In total, survey data from 81 of the 506 eligible Swiss students and 205 of the 750 eligible Turkish students, who had answered at least half of the 23 BERNCA-R items were included in the analyses. The characteristics of these students included are shown in Table 2. The average age of the participating students was 23.1 years in the Swiss sample and 21.6 years in the Turkish sample. The proportion of female students was higher in the Swiss sample (93.8%). >50% of the Swiss students were 'very satisfied' with their career choice in nursing, whereas the majority (64.4%) of Turkish students were 'moderately satisfied'.

**Table 2:** Sample characteristics.

	**Switzerland**	**Türkiye**
**Number of nursing students included in the analysis** – n	81	205
**Age -** mean **±** SD, range	23.1 ± 4.6, 19–54	21.6 ± 1.6, 19–31
**Sex -** n (%)		
Female	76 (93.8)	137 (66.8)
Male	5 (6.2)	68 (33.2)
**Academic year -** n (%)		
First year of study	2 (2.5)	-
Second year of study	51 (63)	79 (38.5)
Third year of study	24 (29.6)	47 (22.9)
Fourth year of study	4 (4.9)	79 (38.5)
**Institution/ Setting -** n (%)		
Acute care hospital	50 (61.7)	186 (90.7)
Children's hospital	7 (8.6)	15 (7.3)
Psychiatric hospital	8 (9.9)	-
Rehabilitation hospital	2 (2.5)	-
Long-term care facility	7 (8.6)	-
District service	7 (8.6)	-
Other	-	4 (2)
**Satisfaction with the choice of nursing career** - n (%)		
Highly satisfied	44 (54.3)	45 (22)
Moderately satisfied	20 (24.7)	132 (64.4)
Dissatisfied	5 (6.2)	20 (9.8)
Highly dissatisfied	12 (14.8)	8 (3.9)

SD: standard deviation.

### 4.2 Implicit rationing of nursing care

Table 3 shows the mean scores of implicit rationing of nursing care, as measured with the BERNCA-R version, in both countries. The overall level of implicit rationing of care reported by Swiss and Turkish nursing students was similar on average (mean 1.12 ± standard deviation 0.55; mean 1.23 ± standard deviation 0.77). Swiss nursing students most often implicitly rationed (rarely to sometimes) the three nursing tasks ‘emotional and psychosocial support’, ‘mobilisation’, and ‘activating or rehabilitating care’, whereas Turkish students most often rationed (rarely to sometimes) the three nursing tasks ‘sponge baths’, ‘partial sponge baths’, and ‘preparation for test and therapies’. Nursing tasks least frequently rationed (never to rarely) in the Swiss sample were ‘change of wound dressing’, ‘monitoring patients as the nurse felt necessary’, and ‘necessary disinfection measures’; tasks least frequently rationed in the Turkish sample ‘necessary disinfection measures’, ‘adequate hand hygiene’ and ‘assessment of newly admitted patients’.

**Table 3:** Comparison of the rationing levels per BERNCA-R items in each country.

**BERNCA-R items**	**Switzerland**			**Türkiye**		
	n	rank	M ± SD	n	rank	M ± SD
1. Sponge baths	69	6	1.28 *±* 0.95	153	**1**	**1.63 *±* 1.15**
2. Partial sponge baths	74	19	0.85 *±* 0.84	163	**2**	**1.52 *±* 1.13**
3. Skin care	73	16	0.99 ± 0.75	180	6	1.41 ± 1.06
4. Oral hygiene	71	10	1.21 ± 1.03	180	9	1.37 ± 1.11
5. Dental hygiene	70	11	1.16 ± 0.99	167	5	1.42 ± 1.10
6. Assist with food intake	66	12	1.11 ± 1.04	165	10	1.34 ± 1.12
7. Mobilisation	74	**2**	**1.74 ± 1.05**	182	12	1.23 ± 1.06
8. Change of the position	69	13	1.10 ± 0.86	185	14	1.16 ± 1.03
9. Emotional and psychosocial support	77	**1**	**1.83 ± 1.03**	195	15	1.15 ± 1.03
10. Necessary conversation	78	8	1.24 ± 0.90	199	20	1.08 ± 1.02
11. Activating or rehabilitating care	69	**3**	**1.50 ± 1.02**	194	11	1.32 ± 1.05
12. Education and training	53	20	0.79 ± 0.88	183	17	1.13 ± 0.95
13. Preparation for discharge	65	14	1.09 ± 0.93	188	8	**1.38 ± 0.97**
14. Monitoring patients as the nurse felt necessary	65	**22**	**0.68 ± 0.93**	199	7	**1.39 ± 1.05**
15. Change of wound dressings	71	**23**	**0.52 ± 0.71**	176	12	1.23 ± 1.03
16. Preparation for tests and therapies	67	16	0.99 ± 0.90	183	**3**	**1.46 ± 1.09**
17. Keep patients who rang waiting	71	6	1.28 ± 1.08	194	4	1.44 ± 1.10
18. Adequate hand hygiene	80	18	0.89 ± 0.99	197	**22**	**0.78 ± 0.99**
19. Necessary disinfection measures	80	**21**	**0.72 ± 0.89**	197	**23**	**0.74 ± 0.97**
20. Studying care plans	78	5	1.38 ± 1.02	199	16	1.14 ± 1.00
21. Assessment of newly admitted patient	68	8	1.24 ± 1.05	199	**21**	**1.07 ± 1.03**
22. Set up care plans	73	4	1.49 ± 1.08	201	19	1.10 ± 1.06
23. Documentation and evaluation of the care	79	14	1.09 ± 0.95	202	17	1.13 ± 1.03

Note. The n varies depending on the item, as only responses on the scale from 0–3 (never=0, rarely=1, sometimes=2, often =3) were included in the calculation of the mean value. The answers 4=not required and 5= is not part of my tasks were excluded. Rank = Ranking number from 1–23, with 1 the most and 23 the least frequently rationed, M = Mean value, SD = Standard deviation.

### 4.3 Prioritisation and workplace characteristics

In the Swiss sample, more than half of the participants indicated that the amount of implicit rationed nursing care in their most recent shift was comparable to that of other shifts. In contrast, in the Turkish sample, the largest proportion of responses (just under 50%) indicated a lower amount compared to other shifts (Table 4).

**Table 4:** Nursing students' opinions on prioritisation strategies, workplace safety, quality of care, and nursing work environment in clinical practice.

**Item /Subscale**	**n**	**Switzerland**	**n**	**Türkiye**
Number of participants -total n	81		205	
**Comparability of the amount of rationed care -** n (%)				
Comparable	76	55 (67.9)	205	82 (40.0)
Lower	76	17 (21.0)	205	100 (48.8)
Higher	76	4 (4.9)		23 (11.2)
**Prioritisation strategies used -** n (%)				
Based on their necessity	63	31 (49.2)	205	151 (73.7)
Based on related consequences for patient safety	63	27 (42.9)	205	18. (8.8)
Based on rules/strategies learned	63	1 (1.6)	205	20 (9.8)
Based on the rules of the current workplace	63	4 (6.3)	205	16 (7.8)
**Number of patients cared for in the most recent shift-** mean **±** SD; median (min-max)	76	4.5 **±** 2.5; 4 (1–16)	205	4.5 **±** 3.2; 3 (1–15)
**Staffing and resource adequacy (PES-NWI-R subscale)^+^-** mean **±** SD				
Staffing and resource adequacy subscale (4 items)	79	2.72 **±** 0.68	205	1.98 **±** 0.63
Enough time	79	2.80 ± 0.72	205	2.11 **±** 0.88
Enough nurses	79	2.47 ± 0.93	205	1.78 **±** 0.76
Enough staff	79	2.78 ± 0.87	205	1.86 **±** 0.79
Adequate support services	79	2.84 ± 0.95	205	2.18 **±** 0.75
**Overall quality of the nursing work environment -** mean **±** SD	78	7.67 **±** 1.66	205	6.56 **±** 2.08
**Patient safety -** mean **±** SD	78	7.31 **±** 1.75	205	6.46 **±** 2.15
**Quality of care -** mean **±** SD	78	7.09 **±** 1.68	205	6.93 **±** 1.76

Notes: The n varies depending on the item, because of missing values, + 4-point Likert-type scale: 1= strongly disagree, 2=disagree, 3=agree, 4= strongly agree.

SD= standard deviation.

Table 4 shows the students' prioritisation strategies and their ratings of workplace characteristics. The most frequently used strategies for rationing of nursing care by Swiss students were ‘based on their necessity’ (49.2%) and ‘based on the associated consequences for patient safety’ (42.9%), whereas Turkish students predominantly prioritised on ‘based on their necessity’ (73.7%). Both Swiss and Turkish students reported caring for a similar number of patients during their most recent shift (mean 4.5 patients). However, Swiss students scored higher on the staffing and resource adequacy subscale, particularly in response to the items ‘enough nurses’ and ‘enough staff’. Although the Swiss students rated patient safety and the quality of care in practice higher than that rated by Turkish students, both groups rated it as average and the ratings were similar overall.

## 5 Discussion

This study aimed to describe and compare the reported levels of implicit rationing of nursing care, prioritisation strategies used, and characteristics of the practice environment as reported by Swiss and Turkish nursing students during their clinical placement. The results of this study showed similar overall rates of implicit rationing of nursing care among Swiss and Turkish nursing students during clinical placement, with marginally higher rates reported in Türkiye. Differences were observed between the two countries in the frequency with which the 23 nursing tasks were rationed and in the prioritisation strategies applied by the students. Swiss nursing students primarily rationed psychosocial and rehabilitative care tasks, whereas Turkish students more often rationed physical and basic daily care tasks. In Türkiye, physical and basic daily care tends to be provided by family members in clinical settings. As a result, tasks such as sponge baths and partial sponge baths are often considered to be outside their primary responsibilities by nursing students. This delegation of care may reflect normalised clinical practice, in which certain care tasks are routinely assigned to family members rather than nursing staff (Guduk and Ankara, 2022). This situation stems from the culturally collectivist structure of Turkish society, which also emphasises being present with patients and being involved in their care. It also reflects the tendency of nurses to compensate for staff shortages by involving patients' family members (Arslan Yürümezoğlu et al., 2023). These findings highlight that the practice settings for students' clinical education represent different approaches to prioritising care. Nevertheless, nursing students in both countries more frequently rationed tasks related to the nurse's independent roles and those aimed at enhancing patients' comfort and well-being than other tasks. These findings align with those reported by Kalánková et al. (2021), who found that nursing students frequently avoided performing tasks associated with the independent role of the nurse, particularly those involving emotional support and patient communication.

Although the concepts of rationed and missed care often produce overlapping empirical findings, distinguishing their theoretical framework is essential. Implicit rationing of nursing care refers to necessary care that is intentionally withheld owing to resource constraints, such as inadequate staffing, limited time, or insufficient skill mix (Schubert et al., 2005). In contrast, missed nursing care involves the omission or delay of required nursing interventions, often associated with issues in individual or systemic performance (Kalisch et al., 2009). Therefore, although the present study is grounded in the rationed care framework, findings from the missed care literature offer valuable complementary insights that help contextualise and enrich the interpretation of the results. Kohanová et al. (2024) demonstrated that nursing students' caring activities, such as providing emotional and psychological support and spending time with patients and family members, were most frequently incomplete. In a qualitative study conducted by Habermann et al. (2022), examples provided by nursing students revealed that basic care needs of patients, such as bathing, dental hygiene, nail and skin care, providing information to patients and relatives, and changing wound dressings, were not being met. Another qualitative study by Kalánková et al. (2021) found that students preferred to delay the fundamental care needs of patients and perceived meeting biological needs as the essence of quality care. Gibbon and Crane, 2018) showed that discharge planning, patient education, mobilisation, and oral hygiene were the most frequently missed areas of care by nursing students.

In contrast to our study, previous research involving nursing students have reported the most frequently omitted nursing tasks as visiting patients without prior announcement, adequate supervision of delegated tasks, evaluating the effectiveness of care, maintaining intake and output documentation, and changing wet clothes (Gibbon and Crane, 2018; Habermann et al., 2022; Kohanová et al., 2024). These differences may be attributed to variations in clinical learning environments, student responsibilities, and institutional expectations. Therefore, identifying these contextual distinctions is essential to develop tailored strategies to reduce rationed care in education and practice. Addressing implicit rationing of nursing care in nursing education requires placing holistic care, a core value of the nursing profession, at the centre of nursing curricula (Abawaji et al., 2024).

Nursing students should adopt a pragmatic approach to the issue and to view missed care as part of the adaptation to the clinical environment (Gibbon and Crane, 2018). However, perceiving missed care as normal may lead to the internalisation of undesirable professional habits. To address this issue, holistic care should be considered as one of the core values of nursing, and must be the focus of curricula. A study in Germany found that student nurses perceived missed nursing care as a violation of patients' rights. For an efficient learning environment, nursing students need time for learning and should not be considered as employees (Habermann et al., 2022). Learning environments should be planned to meet this need, ensuring that missed care is not normalised in clinical settings and does not become a learned professional behaviour. Moreover, such environments equip students with the skills to appropriately prioritise essential interventions when resources are insufficient to meet every patient’s needs.

In this study, the most common prioritisation strategies used by all students to ration care were ‘based on their necessity’ and ‘based on the associated consequences for patient safety’. However, Turkish students more frequently rationed care “based on their necessity”. One of the keys to reducing rationing of care is effective prioritisation. In cases where access to care is limited for various reasons, prioritisation skills are required for the nurse to focus on which patient needs should be addressed through a planned decision-making process (Moradi et al., 2023). This decision-making process is influenced by the nurse's values and the characteristics of the clinical environment (Lake et al., 2009). Nursing students can learn and use prioritisation strategies by being influenced by the values and characteristics of the units and institutions to which they are assigned during clinical placement (Bassi et al., 2025). Although clinical learning environments are essential for prioritisation skills, including educational content and strategies in theoretical classroom training is important to teach this skill (Fernaays, 2024). A high frequency of implicit rationing of nursing care has been reported by nursing students in this study; hence, preparing them for these situations in clinical practice by including this issue and appropriate management strategies in both curricula and clinical placements is crucial. In addition, educators in practice should reflect on these situations with students and discuss the strategies used to organise and prioritise necessary nursing care.

The average number of patients that Swiss and Turkish nursing students were responsible for during their most recent working shifts was 4.5, with similar ranges in both groups (1–16 and 1–15, respectively). Similarly, a study from Slovakia reported that an average of 3.33 student nurses cared for 16.73 patients during their most recent shift (Kohanová et al., 2024). These findings suggest that, although some students in our study were responsible for a relatively high number of patients, the average nurse-to-patient ratio was similar to those reported in international contexts. Considering that studies report nurse-to-patient ratios in clinical settings from approximately 1:5.4 (4.2–7.6) (Lasater et al., 2021) to 1:9.2 (7.4–19.1) (Chau et al., 2025), it is evident that nursing students are also impacted by these high patient loads. Kohanová et al. (2024) identified the number of patients and the perceived competence of the nurse as the most significant determinants of unfinished care.

The staffing and resource adequacy of the practice environments are also determinants of better patient care outcomes (Al-ghraiybah et al., 2024). In this study, Swiss nursing students scored higher (mean 2.72 ± 0.68) than Turkish students (mean 1.98 ± 0.63) on the Practice Environment Scale of the Nursing Work Index sub-scale. Similarly, in a study of nursing students in Spain, the ‘Staff and Resource Adequacy’ sub-dimension was rated at 2.52 points, which was lower than the ratings for the other sub-dimensions (Rodríguez‐García et al., 2021). In the Practice Environment Scale of the Nursing Work Index sub-scale, scores above 2.5 indicate a positive practice environment, whereas scores below 2.5 are considered negative (Lake, 2002). In this study, although Swiss students had a more positive perception of the work environment, it is noteworthy that students in both countries rated patient safety and quality of care at a moderate level. This finding indicates potential areas for improvement in clinical education in both countries. Engaging students in discussions, emphasising that improving patient safety is the primary goal, and seeking their contributions for identifying improvements is vital (Palese et al., 2023). Further to gain a deeper understanding of students’ experiences with rationed nursing care, multi-centre and cross-national studies are needed to explore how clinical care culture and nursing education curricula influence students’ perceptions and behaviours related to rationed nursing care.

## 6 Limitations

This study has several limitations. Firstly, as the data were self-reported by students, it cannot be rule out thar the results were influenced by social desirability bias. Second, students may not always have recognised that they had implicitly rationed the necessary nursing care, which may have led to an underestimation of its frequency. Thirdly, the study was conducted with students from only four universities in Türkiye and Switzerland, which restricts the generalisability of the results. A further limitation is the low response rate and the relatively small resulting sample size. Three reminders were sent to encourage participation and collect reliable data without pressuring respondents.

## 7 Conclusions

The results of this cross-sectional study indicate that implicit rationing of nursing care is a significant issue for bachelor nursing students during their clinical placement. To prepare students for these situations and support them in managing them appropriately, it is recommended to integrate this topic into nursing education curricula and clinical placements, along with training in appropriate prioritisation strategies to manage scarce resources and prioritise necessary nursing care. Additionally, future research is needed to explore the underlying factors contributing to implicit care rationing among nursing students and to identify targeted strategies to mitigate its impact on patient outcomes and professional socialisation.

## Funding sources

This research did not receive any specific grant from funding agencies in the public, commercial, or not-for-profit sectors.

## Declaration of generative AI and AI-assisted technologies in the manuscript preparation process

AI-assisted technologies were used for language editing.

## CRediT authorship contribution statement

**Havva Arslan Yürümezoğlu:** Writing – review & editing, Writing – original draft, Resources, Methodology, Conceptualization. **Milena Marta Bruschini:** Writing – review & editing, Software, Formal analysis, Data curation. **Maria Schubert:** Writing – review & editing, Supervision, Project administration, Methodology, Data curation, Conceptualization.

## Declaration of competing interest

The authors declare that they have no known competing financial interests or personal relationships that could have appeared to influence the work reported in this paper.
